# Influence of the Storage in Bottle on the Antioxidant Activities and Related Chemical Characteristics of Wine Spirits Aged with Chestnut Staves and Micro-Oxygenation

**DOI:** 10.3390/molecules27010106

**Published:** 2021-12-24

**Authors:** Sheila Oliveira-Alves, Sílvia Lourenço, Ofélia Anjos, Tiago A. Fernandes, Ilda Caldeira, Sofia Catarino, Sara Canas

**Affiliations:** 1 Instituto Nacional de Investigação Agrária e Veterinária, Polo de Dois Portos, Quinta de Almoinha, 2565-191 Dois Portos, Portugal; silvia.lourenco@iniav.pt (S.L.); ilda.caldeira@iniav.pt (I.C.); 2 Instituto Politécnico de Castelo Branco, Quinta da Senhora de Mércules, 6001-909 Castelo Branco, Portugal; ofelia@ipcb.pt; 3 CEF—Instituto Superior de Agronomia, Universidade de Lisboa, Tapada da Ajuda, 1349-017 Lisboa, Portugal; 4 Centro de Biotecnologia de Plantas da Beira Interior, Quinta da Senhora de Mércules, 6001-909 Castelo Branco, Portugal; 5 CQE—Centro de Química Estrutural, Associação do Instituto Superior Técnico para a Investigação e Desenvolvimento (IST-ID), Universidade de Lisboa, 1049-001 Lisboa, Portugal; tiago.a.fernandes@tecnico.ulisboa.pt; 6 DCeT—Departamento de Ciências e Tecnologia, Universidade Aberta, Rua da Escola Politécnica, 141-147, 1269-001 Lisboa, Portugal; 7 MED—Mediterranean Institute for Agriculture, Environment and Development, Institute for Advanced Studies and Research, Universidade de Évora, Polo da Mitra, Ap. 94, 7006-554 Evora, Portugal; 8 LEAF—Linking Landscape, Environment, Agriculture and Food Research Center, Instituto Superior de Agronomia, Universidade de Lisboa, Tapada da Ajuda, 1349-017 Lisboa, Portugal; sofiacatarino@isa.ulisboa.pt; 9 CEFEMA—Center of Physics and Engineering of Advanced Materials, Instituto Superior Técnico, Universidade de Lisboa, Av. Rovisco Pais, 1, 1049-001 Lisboa, Portugal

**Keywords:** wine spirit, storage in bottle, antioxidant activity, DPPH assay, FRAP assay, ABTS assay, phenolics

## Abstract

Different ageing technology of wine spirits (WSs) has been investigated, but little has been published on the chemical evolution of aged WS during storage in bottle. The purpose of this study was to examine how 12 months of storage in bottle affected the evolution of antioxidant activity (DPPH, FRAP and ABTS assays), total phenolic index (TPI) and low molecular weight (LMW) compounds content of the WSs aged through alternative technology using three micro-oxygenation levels (MOX) and nitrogen control (N). Results revealed the ability of phenolic compounds from aged WSs to scavenge free radicals during storage in bottle. Among the in vitro antioxidant-activity methods, FRAP assay was the more effective to differentiate WSs according to the ageing technology. Concerning the overall influence of storage in bottle on antioxidant activity, and TPI and LMW compounds content, the higher results were obtained for the MOX modalities (O15, O30 and O60), which showed a similar evolution. In summary, this study provides innovative information, demonstrating that the differences between the aged WSs imparted throughout the ageing process (resulting from different MOX levels) were mostly retained, and only slight modifications during storage in bottle were found.

## 1. Introduction

The alternative ageing technology using wood fragments (staves, tablets, cubes and chips, among others) combined with micro-oxygenation (MOX) applied to beverages stored in stainless steel tanks intends to simulate the ageing process that occurs in wooden barrels, but in a more sustainable way–less time, lower cost and lower environmental impact [1,2]. Studies made on wine spirit (WS) showed that it provides some improvements in the physicochemical and sensory characteristics of beverages during the ageing time [3,4]. Specifically, during the ageing process, the oxygen applied in small amounts plays a crucial role in oxidation, condensation and other reactions involving low molecular weight (LMW) compounds extracted from the distillate, the wood and their derivatives [5,6,7]. As a result, they positively influence the WS colour, aroma and taste, which are determinants of consumer choice. Consumers are becoming more informed and demanding, and, therefore, they also value foods and beverages based on their bioactive chemical content [8]. 

Among the bioactive compounds, phenolic compounds are one of the most important and well-known group of natural antioxidants. In beverages, the antioxidant activity is mainly correlated with phenolic composition [8,9]. The antioxidant activity of phenolics is linked to their protective effects, since they are responsible for the body’s defensive mechanisms against pathologies associated with the attack of free radicals, specifically by reactive oxygen species (ROS) radicals, a condition known as ‘oxidative stress’ [10]. Thus, daily intake of phenolic compounds is involved in the prevention of chronic diseases caused by oxidative stress such as neoplasia, atherosclerosis and neurodegenerative diseases [9,10,11,12]. ROS also plays an important role in cancer development and ischemic stroke complications [13,14]. However, the health benefits of phenolics are mostly dependent on their bioaccessibility and bioavailability, which can be measured regarding the change in the concentration and metabolites of solubilized components in the digestive tract and circulatory system [15,16]. The bioactivity of phenolic compounds from aged WS was previously reported by Kiviniemi et al. [17], showing an increase of plasma antioxidant status in healthy young men after the intake of aged WS. 

In addition, the phenolic compounds of the aged WS are almost exclusively extracted from the wood [18]. The wood botanical species traditionally used in their ageing is Limousin oak (*Quercus robur* L.) wood, but in particular cases the chestnut (*Castanea sativa* Mill) wood has gained prominence due to sustainable aspects [19]. Comparing the LMW phenolic composition of these kinds of wood, chestnut shows higher concentration, particularly of gallic acid [20]. On the other hand, the studies on the traditional technology using wooden barrels revealed that this feature was imparted to the aged WS along with higher antioxidant activity [21,22]. 

In a previous study, in which the WS was aged with wood staves (from chestnut and Limousin oak wood) and a single MOX level (2 mg/L/month), positive and significant correlations between ellagic acid, gallic acid, vanillin and syringaldehyde, and the antioxidant activity assessed by the DPPH method were found [23].

Research is currently in course under the Project Oxyrebrand (https://projects.iniav.pt/oxyrebrand/index.php/pt/ (accessed on 25 November 2021)) by studying the effect of three levels of MOX or nitrogen (control) applied to the same WS kept in 50 L glass demijohns with chestnut wood staves inside. Since the antioxidant activities of the bioactive compounds are affected by their chemical structure, oxidation conditions and concentration [9], it is necessary to investigate the effect of different levels of oxygen applied through MOX on the antioxidant activities of aged WSs. 

**Figure 1:**
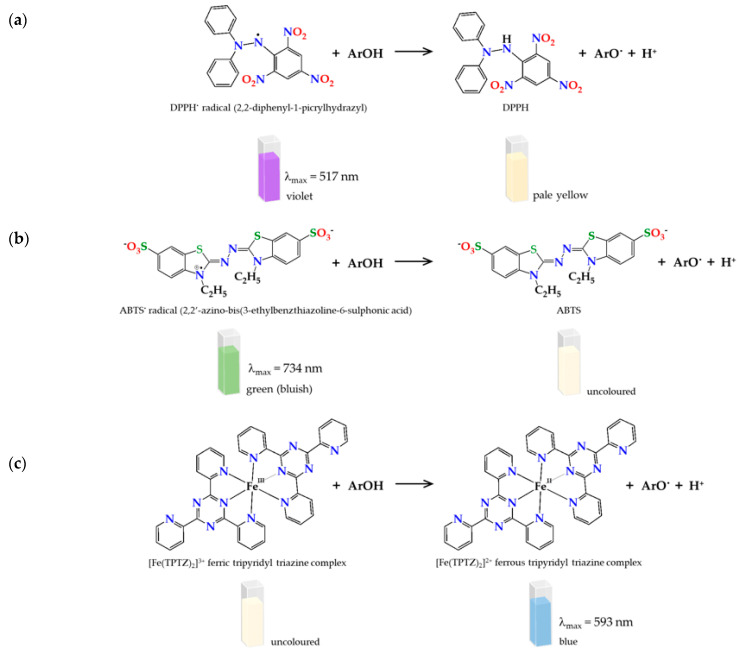
Radical scavenging reaction mechanism for (**a**) 2,2-diphenyl-1-picrylhydrazyl (DPPH), (**b**) 2,2′-azino-bis(3-ethylbenzothiazoline-6-sulfonic acid) (ABTS), and (**c**) Ferric Reducing Antioxidant Power (FRAP).

For this purpose, in vitro methods can be applied, allowing the determination of the antioxidant activity based on the radical scavenging properties. Radical scavenging assay includes methods based on hydrogen atom transfer (HAT) or single electron transfer (SET) mechanisms [9]. Spectrophotometric SET-based assays measure the capacity of an antioxidant in the reduction of an oxidant and are faster and cheaper (colorimetry methods) than HAT [24]. 2,2-diphenyl-1-picrylhydrazyl (DPPH), 2,2’-azino-bis(3-ethylbenzothiazoline-6-sulfonic acid) (ABTS) and Ferric Reducing Antioxidant Power (FRAP) assays represent main SET-based methods. They produce comparable results for the antioxidant activity given that the method approach is almost the equivalent: an antioxidant reaction with an organic radical (DPPH and ABTS assays) or with a Fe(III) complex (FRAP assay) [25,26]. Besides, they are complementary, and thus more reliable/robust results on the antioxidant activity are attained (Figure 1).

In this project, despite the interesting results attained on some physicochemical and sensory features of the WSs during the ageing process [4,6,27], it is imperative to assess the overall quality of the aged WSs during the storage in bottle in order to select the best MOX strategy. Indeed, through this innovative approach, the intention is to gain a better understanding of the ageing chemistry, determine whether the characteristics imparted by the ageing modality are retained throughout storage in bottle, whether they improve or whether they persist. Some factors, including the closure, exposure to light, temperature, bottle position and the availability of oxygen in headspace height, may affect the characteristics of the aged WS during this stage [25]. Thus, this study aims to investigate, for the first time, the influence of the storage in bottle over 12 months on the evolution of antioxidant activities (DPPH, FRAP and ABTS assays), total phenolic index and low molecular weight compounds concentrations (HPLC) of the WSs aged through the four ageing modalities (three MOX level and control), and to examine the correlation of these characteristics as well.

## 2. Results and Discussion

### 2.1. Effect of the Storage Time in Bottle on the Antioxidant Activities of the Aged WS

The results regarding the method’s donating capacity of electrons (SET) provide important information on their intrinsic antioxidant potential with minimum environmental interference [24]. It is crucial to emphasize that in vitro antioxidant activity should not be performed on the basis of a single method due to the differences between the assay systems available [28]. For this reason, to assess the antioxidant activity of the aged WSs during the storage in bottle, three specific in vitro assays (DPPH, FRAP and ABTS assays) were used, which showed a good accuracy, reproducibility and easy measurement [24].

**Figure 2:**
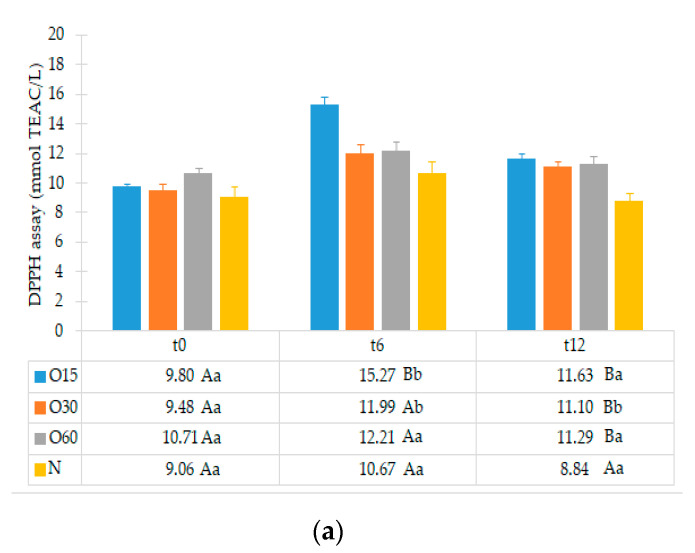

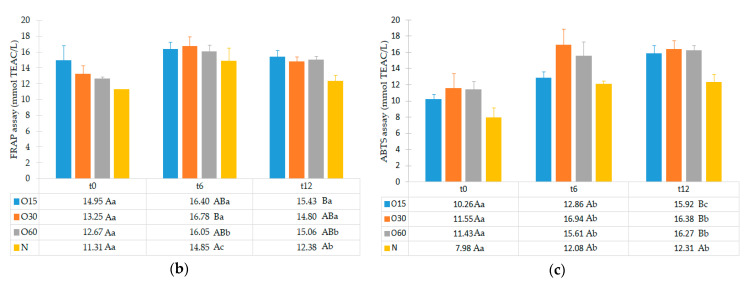
Average values of antioxidant activity of aged WS in each storage time (0, 6, 12 months) according to ageing modalities (O15, O30, O60 and N) using single electron transfer (SET) method: (**a**) DPPH assay; (**b**) FRAP assay; (**c**) ABTS assay. Results are expressed as mean values ± standard deviation (*n* = 4). For each analytical determination: different uppercase letters (A, B) in the same column denote significant differences between ageing modalities in each storage time by Tukey’s test (*p* < 0.05); different lowercase letters (a, b, c) in the same row denote significant differences between storage times for each ageing modality by Tukey’s test (*p* < 0.05).

In addition, little is known about the changes in the antioxidant activity of aged WS that occur during storage in bottle compared to the initial storage time (end of the ageing process). Figure 2 depicts the antioxidant activities of the aged WSs from the four ageing modalities (O15, O30, O60, N) assessed by the DPPH, FRAP and ABTS methods during storage time (0, 6, 12 months) in bottle. 

The statistical analysis revealed that the antioxidant activities of the WS from the four ageing modalities assessed by the DPPH, FRAP and ABTS assays were not significantly different in the beginning of storage (t0). Little difference in antioxidant activity values (DPPH, FRAP and ABTS assays) between the ageing modalities may be ascribed to the MOX level and the wood variability (even with the same surface to volume ratio used). 

After six months in bottle (t6), an increase in the antioxidant activities by DPPH, FRAP and ABTS assays for the WSs resulting from the four ageing modalities was observed. This increase was significantly higher in O15 and O30 by DPPH assay, in O60 and N by FRAP assay, and in all modalities by ABTS assay. In addition, the O15 WS exhibited significantly higher antioxidant activity by DPPH assay than the WSs from other ageing modalities, whereas the O30 had higher antioxidant activity by FRAP assay. These changes in the antioxidant activities are presumably due to differential transformation of phenolic compounds into polymeric and condensed forms, under different oxygen content resulting from the MOX levels combined with the action of factors ruling the storage in bottle (light, temperature, oxygen transfer from the cork, among others). Indeed, these compounds exhibit different chemical properties and reactivity towards the organic radical reagent (DPPH and ABTS assays) and with a Fe(III) complex (FRAP assay) [29], according to their degree of hydroxylation and extent of conjugation [30,31]. In red wines, the increase of polymeric phenolics was also evidenced during the storage in bottle [25,32,33,34]. Furthermore, synergistic and/or antagonistic phenomena among these compounds and/or with other compounds present in aged WSs may also contribute to such differences [23,35]. 

On the other hand, at t6 the FRAP assay showed higher antioxidant activity values than DPPH and ABTS assays for the studied WSs, whereas the last ones displayed similar values. These results are in accordance with those of Riviero-Pérez et al. [29] for red wine. Likewise, Mrvčić et al. [36] reported that the antioxidant activities of wine brandies and homemade rum were higher by FRAP assay than by DPPH assay. 

After 12 months in the bottle (t12), the antioxidant activity by the DPPH and FRAP assays decreased significantly only in O15 WS and control WS, respectively. On the contrary, the antioxidant activity of O15 WS increased significantly by ABTS assay. Comparing the WS from the four ageing modalities at this storage time, significantly lower antioxidant activity (DPPH, FRAP and ABTS assays) in the control (N) was observed, while the MOX modalities behaved similarly. The oxidation and polymerization reactions of LMW compounds during storage may lead to changes in the antioxidant activity of the aged WS, as a consequence of changes in the chemical composition mentioned above [30,37] and redox balance [25,38].

Regarding the average results of the three assays performed, DPPH assay showed the lowest values at t12. This behaviour can be ascribed to the reaction mechanism implied in the DPPH assay, in which many antioxidants may react with different kinetics or may not react at all [39]. Furthermore, the reversibility of reaction between DPPH and antioxidants may not be entire [39,40]. Accordingly, the reversibility of the reaction may lead to low measurement of antioxidant activity of many compounds [39,41].

Considering all results, the current study suggests that the FRAP assay was the more effective method for differentiating WSs, based on the ageing modality, during storage in bottle. It also reveals the efficiency of phenolic compounds from the aged WSs to scavenge free radicals during storage in bottle, suggesting that these bioactive compounds are preserved after the ageing process (regardless of the MOX modality applied) and may have positive health benefits for the consumers. Similar results were reported in previous studies using the same in vitro methods, demonstrating that the antioxidant activity of white and red wines increased during storage in bottle [25,38]. 

### 2.2. Relationship between the Antioxidant Activity and Phenolic Content of the Aged WS

**Figure 3:**
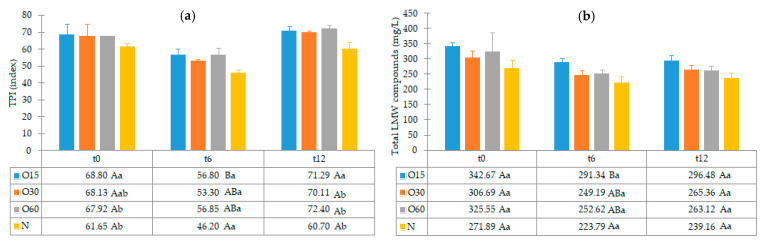
Average values of total phenolic index (TPI) and total LMW compounds in each storage time (0, 6, 12 months) according to ageing modalities (O15, O30, O60 and N): (**a**) TPI values; (**b**) Total LMW compounds content (calculated by summing the mean values of all compounds determined by HPLC method). Results are expressed as mean values ± standard deviation (*n* = 4). For each analytical determination: different uppercase letters (A, B) in the same column denote significant differences between ageing modalities in each storage time by Tukey’s test (*p* < 0.05); different lowercase letters (a, b) in the same row denote significant differences between storage times for each ageing modality by Tukey’s test (*p* < 0.05).

The antioxidant activity of the aged WS, which has been clearly demonstrated in several studies, is mostly assigned to its phenolic composition [22,23,42]. Figure 3 depicts the average values of TPI and total LMW compounds in each storage time (0, 6, 12 months) according to the ageing modalities (O15, O30, O60 and N). 

TPI and LMW compounds contents were not significantly different between the WSs from the four modalities at t0. Therefore, the different MOX flow rates applied during the ageing process with chestnut wood staves did not promote significant differences in the phenolic content between the ageing modalities (O15, O30 and O60), as observed at the end of the ageing process [27]. These results may explain the similar antioxidant activity attained by DPPH, FRAP and ABTS methods at this storage time (Figure 2).

**Figure 4:**
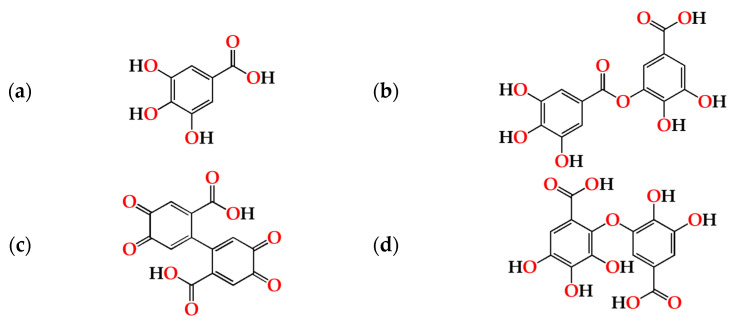
Chemical structures of (**a**) gallic acid, (**b**) digallate (digallic acid) formed by esterification, (**c**) C-C dimer formed by gallic acid autoxidation, (**d**) C-O dimer formed by gallic acid oxidation.

Regarding the evolution over the storage time, the total LMW compounds content did not change significantly regardless of the ageing modality (O15, O30, O60 and N). The TPI of the aged WSs decreased after 6 months of storage in bottle for all ageing modalities, showing significant differences for O30, O60 and N, and then increased up to 12 months. Moganas et al. [32] reported a similar decrease of total phenolic during the storage of red wine in bottle, which was assigned to the transformation of LMW phenolics into condensed forms. Moreover, the behaviour observed in the present study suggests that lower TPI values and the increase of antioxidant activity observed after 6 months of storage in bottle (Figure 2) may be correlated with the autoxidation, esterification or polymerization of gallic acid [25]. Several studies reported that the antiradical efficiency of phenolic compounds tends to increase in the order of their progressive polymerization [43,44,45]. Tulyathan et al. [46] found that the dimerization of gallic acid occurs during oxidation, giving rise to carboxylic acids, indicating that the gallic acid dimers, such as digallate, C-O or C-C dimer, are spontaneously formed in an acidic medium (Figure 4). According to Pan et al. [47], higher antioxidant activity of gallic acid dimer than gallic acid monomer is presumably due to the valency effects. Gallic acid’s excellent antioxidant action is due to a number of other variables, including its planar geometry, low binding energy of hydroxyl groups, electron-withdrawing of carboxylic groups at *para* position, electron-donors at both *ortho* positions and stabilization via two intermolecular hydrogen bonds with *ortho* hydroxyl groups [48]. Indeed, gallic acid is a stronger radical scavenger and seems to have an indirect effect over the other phenolics present in aged WSs [49,50]. This hypothesis explains the depletion of gallic acid equally for all ageing modalities after 6 months of storage, showing a decrease of 33% for O15 modality and 39% for O30, O60 and N modalities (Table 1). Burin et al. [25] also reported a reduction of 50% of gallic acid content in red wines after 8 months of storage in bottle.

The slight changes observed in the LMW compounds and antioxidant activities from 6 to 12 months of storage in bottle may be ascribed to the lower level of available oxygen (uptake from headspace and/or dissolved into the aged WS). However, the significant increase of the TPI values found for all WSs may derive from the hydrolysis of phenolic glycosides and hydrolyzed tannins giving rise to free forms, causing this effect [22,50]. 

It should be noted that in each storage time, the TPI and total LMW compounds content of the control WS (N) were slightly lower than the those of WS from MOX modalities (O15, O30 and O60), which is in line with the pattern observed throughout the ageing experiment [6,27]. These findings are in agreement with the lower antioxidant activity levels found in the control WS. 

In summary, the results of antioxidant activity evolution over time were coherent with those of phenolic composition obtained for the ageing modalities. According to the correlation analysis, a positive and significant correlation between the results of the antioxidant activity determined by three methods and TPI exist: r_ΣDPPH_ = 0.88; r_ΣFRAP_ = 0.94 and r_ΣABTS_ = 0.89. Furthermore, total LMW compounds also showed a significant correlation with values of the antioxidant activity by FRAP assay (r _ΣFRAP_ = 0.77) and DPPH assay (r_ΣDPPH_ = 0.72); however, no correlation was obtained with the ABTS assay. The higher correlations found between TPI, DPPH, FRAP and ABTS assays suggest that synergy between different phenolic compounds (phenolic acids, tannins, coumarins, among others) determine the antioxidant activity of the aged WS, as reported by Nocera et al. [23].

The evolution of LMW compounds was assessed individually over the storage in bottle (Table 1). These compounds exist in free or conjugated forms in the wood staves and are also derived from the thermal decomposition of lignin, gallotannins and ellagitannins during cooperage heat treatment [6,51]. 

Among the quantified LMW phenolic acids, gallic acid, followed by ellagic acid, was the most abundant in the aged WS from the four modalities, whereas ferulic acid had the lowest concentration (Table 1). Gallic acid content in aged WS depends on the botanical species and toasting level of the wood staves, hydrolysis of wood digallic acid and gallotannins [18,52]. According to previous studies [2,6,53], gallic acid can act as an ageing marker of chestnut wood, allowing evaluate the authenticity of WS aged with this botanical species. There was an overall decrease of gallic acid contents of the aged WSs in the four modalities after 6 months in bottle.

Concerning ellagic acid in aged WS, the ellagitannin degradation during the heat treatment of the wood staves and ellagitannin hydrolysis during the ageing process are the major sources [54]. Also, vanillic acid can be directly extracted from chestnut wood staves or be formed by oxidation of vanillin during the ageing time, whereas syringic acid is formed during toasting by the oxidation of syringaldehyde [55]. According to Table 1, no significant changes were observed in ellagic, syringic and vanillic acids contents of the aged WS during the storage time (0, 6 and 12 months), indicating that, in general, the storage in bottle did not affect these phenolic acids. Conversely, ferulic acid content did not vary for up to 6 months but showed a sharp decrease between 6 and 12 months of storage in bottle for all ageing modalities. These results are in agreement with those reported by Revilla et al. [56] and Burin et al. [25] for wines stored in bottle. 

Regarding the phenolic aldehydes, they are mainly produced by thermal degradation of the terminal monomer units of lignin, and the cinammic aldehydes can also give rise to benzoic aldehydes during the heat treatment in cooperage [52,57]. In addition, their presences in the WSs during ageing have also been ascribed to the lignin hydroalcoholysis [6]. The results showed that no significant changes occurred in the contents of coniferaldehyde and sinapaldehyde in the WSs from the four modalities over the storage time in bottle (Table 2). Interestingly, vanillin and syringaldehyde contents of the aged WSs showed a slight increase during this stage. 

Thus, the storage in bottle had low impact on the aged WSs phenolic profile. The low concentrations exhibited by the WS from control modality (N) are in accordance with the results attained at the end of the ageing process [6], confirming the importance of MOX application combined with wood staves during the ageing. 

Table 3 shows the average values of furanic aldehydes contents in each storage time (0, 6, 12 months) according to the ageing modalities (O15, O30, O60 and N). Furanic aldehydes are present in the wood staves as a result of hemicelluloses and cellulose degradation during the heat treatment in cooperage [58]. In the aged WSs, the major components assessed in this group were furfural, 5-methyl furfural and 5-hydroxymethyl furfural (HMF). Among the furanic aldehydes, furfural is the most plentiful compound due to its predominance in the wine distillate and aged WSs [6]. Furfural, HMF and 5-methylfurfural are important because they confer a caramel and toasted-almond-like aroma to the aged WS [59]. Regardless of the ageing modalities, similar evolution patterns were observed for furfural and HMF in the WSs during the storage in bottle; no significant differences from t0 to t12 were found, except for the control WS (N), in which a significant decrease occurred after 6 months of storage. The behaviour of 5-methylfurfural was quite different, showing a significant decrease for all modalities in the first six months of storage and subsequently tended to stabilize in all WSs. This depletion may have occurred due to the oxidation of 5-methyl furfural to 5-methyl-2-furonic acid [60,61].

Regarding the general effect of storage in bottle on the antioxidant activity, TPI and LMW compounds content of the aged WSs, similar evolution of the WSs from the MOX modalities (O15, O30 and O60) was observed. In addition, differences between the aged WSs imparted during the ageing process (resulting from different MOX levels) were mostly preserved. 

### 2.3. Multivariate Analysis.

**Figure 5:**
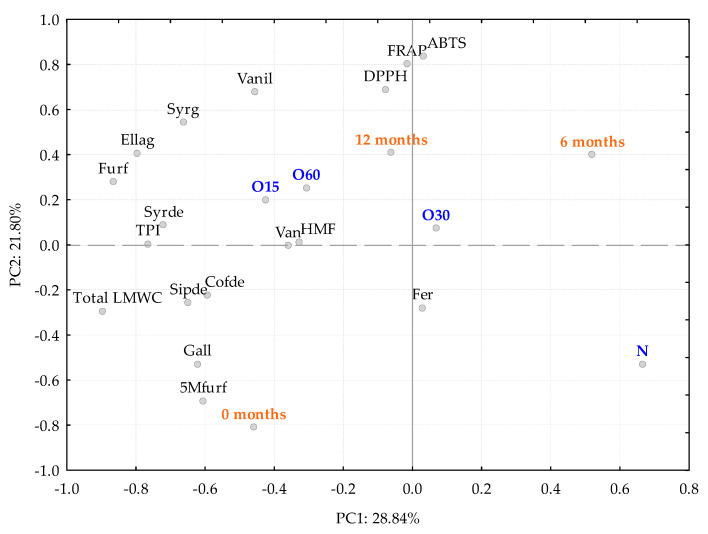
Principal Components Analysis (standardized scores and loadings)) with results of antioxidant activity, phenolic content (TPI) and individual LMW (Low molecular weight) compounds from aged WS using ageing modalities over time storage in the bottles. TPI—total phenolic index; Gall—gallic acid; Ellag—ellagic acid; Van—vanillic acid; Syrg—syringic acid; Fer—ferulic acid; Vanil—vanillin; Syrde—syringaldehyde; Cofde—coniferaldehyde; Sipde—sinapaldehyde; Furf—furfural; HMF—5-hydroxymethylfurfural; 5Mfurf—5-methylfurfural.

Principal Component Analysis (PCA) was used to study the overall effect of the storage in bottle on the aged WSs’ characteristics (antioxidant activities, TPI and LMW (Figure 5). The results were separated by the two components (PC1 and PC2), which only explained 50.65% of the total variability. PC1 (28.84%) revealed a weak influence of the storage time in the chemical composition of the WSs from different ageing modalities. PC2 accounted for 21.80% of the variation associated with the ageing modalities with oxygen (MOX) and without it (N). This difference between MOX modalities and control modality was already observed at the end of the ageing process [6,27]. 

In summary, the percentage of variation explained by PC1 and PC2 is low, indicating that there is no evident separation of the WSs according to the storage in bottle, corroborating the results of the analysis of variance. Similar to at the end of ageing [6,27], the results suggest a slightly higher performance of O60 in terms of antioxidant activity and LMW compounds content, followed by O15 and a more pronounced differentiation from O30 and N.

## 3. Materials and Methods

### 3.1. Chemical and Reagents

Formic acid (98% *v*/*v*, analytical grade) and methanol (99.9% *v*/*v*, LC gradient grade) were purchased from Merck (Darmstadt, Germany), and ethanol (99.9% *v*/*v*, LC gradient grade) was purchased from Carlo Erba (Val de Reuil, France). The ultrapure water (conductivity < 0.055 µS/cm) and distilled water (conductivity < 6.0 µS/cm) were obtained from Arium Comfort System (Sartorius, Goettingen, Germany). Trolox (6-hydroxy-2,5,7,8-tetramethyl-chromane-2-carboxylic acid), TPTZ (2,4,6-tris(2-pyridyl)-s-triazine), ABTS (2,2′-azino-bis (3-ethylbenzothiazoline-6-sulfonic acid) and sodium acetate trihydrate were purchased from Sigma-Aldrich (Steinheim, Germany). DPPH (2,2-diphenyl-1-picrylhydrazyl) radical was purchased from TCI (Tokyo, Japan). Potassium persulfate (K_2_S_2_O_8_) and iron(III) chloride hexahydrate (FeCl_3_·6H_2_O) were purchased from Honeywell Fluka (Seelze, Germany). Gallic acid monohydrate (gal), ellagic acid dehydrate (ellag), vanillic acid (van), syringic acid (syr), ferulic acid (ferul), furfural (furf), 5-hydroxymethylfurfural (HMF), 5-methylfurfural (5mfurf), and vanillin (vanil) were purchased from Fluka (Buchs, Switzerland). Syringaldehyde (syrde), coniferaldehyde (cofde), sinapaldehyde (sipde) and 4-hydroxybenzaldehyde were purchased from Sigma-Aldrich (Steinheim, Germany). All standard compounds had purity > 97%. The standard solutions were freshly prepared prior to use with ethanol/water (75:25, *v*/*v*). 

### 3.2. Experimental Design and Aged WSs Sampling

The experiment comprised two phases (Table 4): 

(1) Ageing trial-was carried out in 50 L glass demijohns (pilot scale), encompassing four ageing modalities: one control modality with nitrogen application, and three MOX modalities (O15, O30, O60), with two replicates. The corresponding experimental design was described in detail by Canas et al. [6]. Briefly, Portuguese chestnut (*Castanea sativa* Mill.) staves manufactured by J. M. Gonçalves cooperage (Palaçoulo, Portugal) with medium plus toasting level were inserted into the demijohns of all modalities and reproducing the surface area to volume ratio of a 250 L barrel. The same wine distillate (alcohol strength, 78.3 *v*/*v*; pH, 5.33; total acidity, 0.12 g acetic acid/L of absolute ethanol; volatile acidity, 0.09 g acetic acid/L of absolute ethanol) produced by Adega Cooperativa da Lourinhã (Lourinhã, Portugal) was used to fill the demijohns. MOX was applied to the WS during the ageing time, providing pure oxygen (X50S Food, Gasin, Portugal) through a multiple diffuser micro-oxygenator (VISIO 6, Vivelys, France) with ceramic diffusers, at different flow rates according to the ageing modality (O). In the control modality (N), pure nitrogen (X50S Food, Gasin, Portugal) was applied continuously over the ageing time through a specific device (Gasin, Portugal) to the WS aiming to decrease the dissolved oxygen as much as possible, thus acting as a control. The eight experimental units were stored in the cellar of Adega Cooperativa da Lourinhã in similar environmental conditions.

(2) Storage in bottle-after 365 days of ageing, the eight aged WS were bottled in the same day in 750 mL amber glass bottles (two bottles from each demijohn), assuring the same level of WS in each bottle. Indeed, the headspace was set at 9.8 mL of air in all bottles to assure that the oxygen ingress into the aged WS was similar, so that the oxidation [62] promoted was controlled and the main effects observed were due to the ageing modality and the storage time. The cork stoppers were sealed with parafilm (Parafilm^®^, Bemis Company, Neenah, WI, USA) to avoid evaporation. The bottles were transported in the same day and stored in the cellar of INIAV-Dois Portos at 19 °C and 80% relative humidity for 12 months. Sampling was carried out at 0, 6 and 12 months. A total of 48 samples (4 modalities × 2 replicates × 2 sampling bottles × 3 storage times) of WS were taken and analyzed to determine the antioxidant activities, total phenolic index and low molecular weight composition, and their correlations as well.

### 3.3. In Vitro Antioxidant Activity Analyses

#### 3.3.1. DPPH Assay

The DPPH (2,2-diphenyl-1-picrylhydrazyl) free radical scavenging activity was performed using the modified method described by Nocera et al. [23]. Briefly, 10 µL of aged WS was added to 3 mL of 8.5 × 10^−5^ M DPPH methanolic solution in a glass tube wrapped with aluminum foil. The tube was vortexed for 10 s and then immediately placed in a water bath (Selecta, Digiterm 3000542, Barcelona, Spain) at 30 ± 1 °C for 60 min; the tube was shaken every 10 min and placed back in the water bath. The absorbance was measured at a wavelength of 515 nm using a Varian Cary 100 Bio spectrophotometer (Santa Clara, CA, USA). The control absorbance (10 µL de methanol and 3 mL of 8.5 × 10^−5^ M DPPH methanolic solution) was measured at the beginning and end of the assay. Trolox was used as a reference standard curve (1–15 mM TEAC) and the results were expressed as mmol Trolox equivalent antioxidant capacity (TEAC)/L of WS. The analyses were performed in triplicate.

#### 3.3.2. ABTS Assay

The ABTS assay was based on the method reported by Rufino et al. [63] with some modifications. The ABTS^•+^ radical cations were prepared by reacting 4 mL of a 7 mmol/L ABTS stock solution with 70.4 μL of 140 mmol/L potassium persulfate solution for 16 h, at room temperature and in the dark. Thereafter, the ABTS solution was diluted by adding ethanol (99.5%) to the ABTS^•+^ radical solution until measured absorbance reached 0.700 ± 0.02 at 734 nm using a Varian Cary 100 Bio spectrophotometer (Santa Clara, CA, USA). The WSs were diluted with absolute ethanol (1:50 *v*/*v*). 3 mL of ABTS solution was added to 30 µL of sample or 30 µL of standard solution, mixed and placed in the water bath (Selecta Digiterm 3000542, Barcelona, Spain) for 6 min at 30 °C. After reaction, the absorbance was measured at 734 nm at room temperature. The control absorbance (30 µL of absolute ethanol plus 3 mL of ABTS solution) was measured at the beginning and end of the assay. Trolox standard curve (0.08–2.0 mM TEAC) was used as reference antioxidant. Results were expressed as mmol Trolox equivalent antioxidant capacity (TEAC)/L of WS. The analysis was carried out in triplicate.

#### 3.3.3. FRAP Assay

The ferric reducing ability was determined by the FRAP (Ferric Reducing Antioxidant Power) method, with modifications [30,64]. The FRAP reagent was prepared with 75 mL of a 0.3 M sodium acetate buffer (pH 3.6), 7.5 mL of 10 mmol/L TPTZ (2,4,6-tris(2-pyridyl)-s-triazine) in a 40 mmol/L HCl solution, plus 7.5 mL of 20 mmol/L FeCl_3_·6H_2_O in the dark. The WSs were diluted with absolute ethanol (1:50, *v*/*v*). Sample or standard solutions, distilled water and FRAP reagent (90 µL sample or standard solution, 270 µL distilled water plus 2.7 mL FRAP reagent) were mixed and kept in a water bath (Selecta, Digiterm 3000542, Barcelona, Spain) for 30 min at 37 °C. After cooling to room temperature, absorbance was measured at 595 nm using a Varian Cary 100 Bio spectrophotometer (Santa Clara, CA, USA). The control absorbance (90 µL ethanol, 270 µL distillated water plus 2.7 mL FRAP reagent) was measured at the beginning of the assay. A Trolox standard curve was also prepared (0.08–1.5 mM TEAC). Results were expressed as mmol Trolox equivalent antioxidant capacity (TEAC)/L of WS. The assay was performed in triplicate.

The study of linearity of Trolox standard curves used in the antioxidant activity assays (DPPH, FRAP and ABTS) was performed using Minitab version 14 (State College, PA, EUA) and showed that the linear regression was the best model for establishing a relationship between the percentage of DPPH inhibition/or ABTS inhibition/or FRAP reduction) and Trolox concentration (mM) (data not shown).

### 3.4. Total Phenolic Index Determination

The total phenolic index (TPI) of the WSs was determined according to Cetó et al. [65]. Briefly, WSs were diluted with distilled water and the absorbance was measured directly at 280 nm using a Varian Cary 100 Bio spectrophotometer (Santa Clara, CA, USA) with a 10 mm quartz cuvette. TPI value of each sample was calculated by multiplying the measured absorbance by the dilution factor. The analyses were performed in triplicate.

### 3.5. Low Molecular Weight Composition Determination

Low molecular weight (LMW) compounds of aged WSs were quantified according to the method of Canas et al. [66]. Chromatography separation of compounds was performed using a HPLC Lachrom Merck Hitachi system (Merck, Darmstadt, Germany) equipped with a quaternary pump L-7100, a column oven L-7350, a UV-Vis detector L-7400, and an autosampler L-7250, coupled with HSM D-7000 software (Merck, Darmstadt, Germany) for management, acquisition and treatment of data. A LiChrospher RP 18 (5 μm, 250 mm × 4 mm ID) column (Merck, Darmstadt, Germany) was used as a stationary phase. The mobile phase consisted of water/formic acid (98:2 *v*/*v*) as eluent A, and methanol/water/formic acid (70:28:2 *v*/*v*/*v*) as eluent B, at a flow rate of 1 mL/min and column temperature of 40 °C. All solvents were filtered through a 0.45 µm PVDF membrane (Cronus filter, Gloucester, UK) prior to analysis. The auto sampler’s temperature was set at 18 °C and the injection volume was 20 µL. Samples were spiked with an internal standard (20 mg/L of 4-hydroxybenzaldehyde). The elution program was as follows: 0–3 min at 0% isocratic B; 3–25 min from 0% to 40% B; 25–43 min from 40% to 60% B; 43–55 min at 60% isocratic B; 55–60 min from 60 to 80% A; 60–65 min at 80% isocratic B; 65–75 min from 80 to 0% B, and finally returning to the initial conditions. Detection was made at 280 nm for phenolic acids (gall, van, syrg, fer and ellag acids) and furanic aldehydes (HMF, furf and 5mfurf), and at 320 nm for phenolic aldehydes (vanil, syrde, cofde and sipde). Quantification of these compounds was performed through calibration curves (mg/L).

### 3.6. Statistical Analysis

Data of antioxidant activities, TPI and low molecular weight composition were expressed as mean ± standard deviation of independent duplicates. One-way analysis of variance (ANOVA) was applied to assess the effects of the ageing modalities on the antioxidant activities, and the TPI and LMW compound contents of the aged WSs for each storage time in bottle. Another one-way ANOVA was carried out to assess the significance of the antioxidant activities, and the TPI and LMW compounds over storage time in bottle. Tukey’s test was made to compare the average values when a significant difference (*p* < 0.05) was found. The correlation between antioxidant activities, and TPI and LMW compound concentrations were determined through the Pearson’s correlation coefficient test, considering a confidence level of 95% (*p* < 0.05). Principal component analysis (PCA) of results was used to evaluate the possible grouping of total LWS compounds for four modalities during the storage time in bottle. Statistical analysis and PCA were performed using Statistica version 7.0 (StatSoft Inc., Tulsa, OK, USA). The results of scores and loadings are standardized and present in the same graphic that identifies the influence of each factor.

## 4. Conclusions

This study provides innovative information on the influence of storage in bottle on the antioxidant activities and phenolic composition of the wine spirits aged with chestnut staves and micro-oxygenation. The results demonstrated that after six months in bottle, an increase in the antioxidant activities and a decrease of total phenolic index for the wine spirits resulting from the four ageing modalities was observed, likely due to the transformation of phenolic acids into polymeric and condensed forms. After 12 months in the bottle, the antioxidant activity of aged wine spirits from the micro-oxygenation modalities behaved similarly, while the control (N) had significantly lower antioxidant activity and TPI values. Therefore, the differences between the aged wine spirits imparted by the ageing process (resulting from different oxygenation levels) were mostly retained. Slightly higher performance of the micro-oxygenation modality with higher oxygen application is in accordance with our previous studies, reinforcing that this technological option seems to be the most suitable for wine spirit quality and ageing sustainability.

## Figures and Tables

**Table 1:** Evolution of the phenolic acids from aged WS in each storage time (0, 6, 12 months) according to ageing modalities (O15, O30, O60 and N).

		Time (Months)			
Phenolic Acids (mg/L)	MOX	0	6	12	
Gall	O15	123.13 ± 16.62 Ab	81.64 ± 13.06 Ba	81.86 ± 10.36 Ba	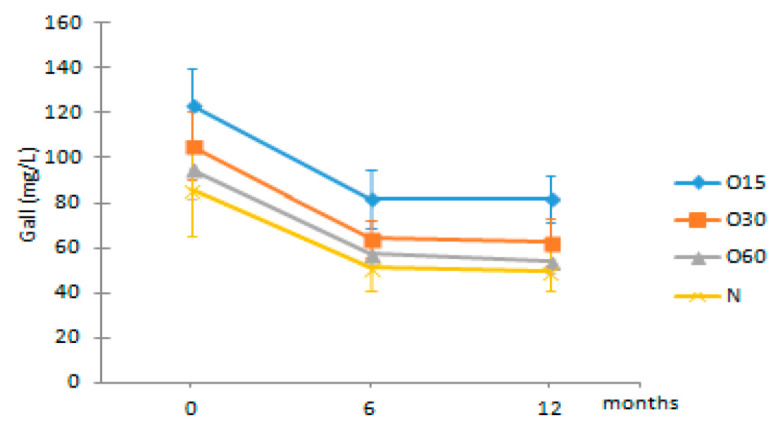
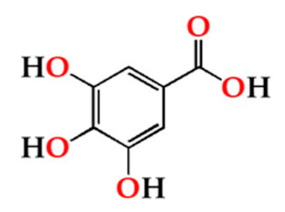	O30	105.52 ± 14.81 Ab	64.21 ± 8.15 ABa	62.58 ± 10.60 Aa	
	O60	94.89 ± 12.99 Ab	57.28 ± 5.22 Aa	54.10 ± 5.45 Aa	
	N	85.69 ± 20.08 Ab	51.56 ± 10.14 Aa	50.01 ± 9.25 Aa	
Van	O15	17.53 ± 7.18 Aa	15.71 ± 4.99 Aa	17.42 ± 5.90 Aa	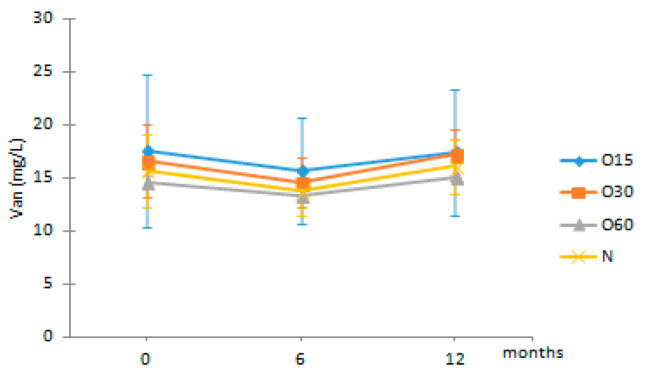
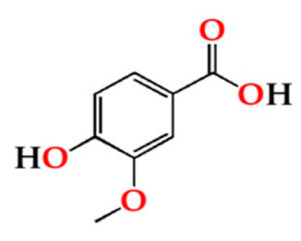	O30	16.63 ± 3.46 Aa	14.55 ± 2.38 Aa	17.18 ± 2.41 Aa	
	O60	14.59 ± 0.61 AAb	13.34 ± 0.06 Aa	15.01 ± 0.14 Ab	
	N	15.66 ± 3.42 Aa	13.75 ± 2.25 Aa	16.07 ± 2.58 Aa	
Ellag	O15	22.71 ± 2.45 Aa	21.71 ± 1.71 ABa	21.73 ± 2.29 ABa	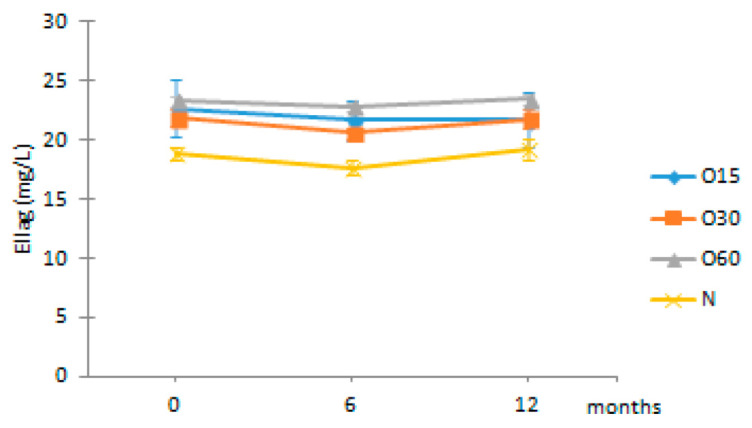
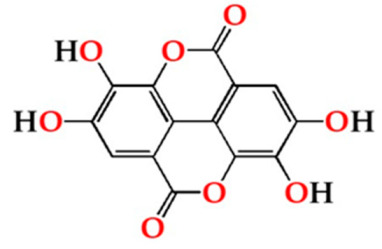	O30	21.89 ± 0.67 Aa	20.63 ± 0.42 Ba	21.82 ± 0.86 ABa	
	O60	23.29 ± 0.48 Aa	22.81 ± 0.37 Ba	23.44 ± 0.44 Ba	
	N	18.93 ± 0.53 AAb	17.70 ± 0.62 Aa	19.30 ± 0.89 Ab	
Fer	O15	1.53 ± 0.79 Ab	1.66 ± 0.67 Ab	0.37 ± 0.11 Aa	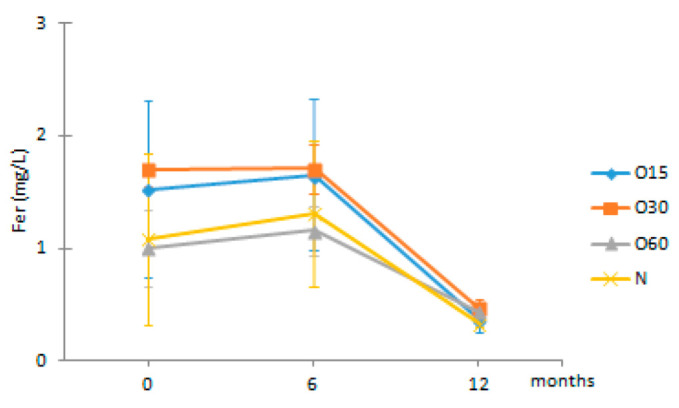
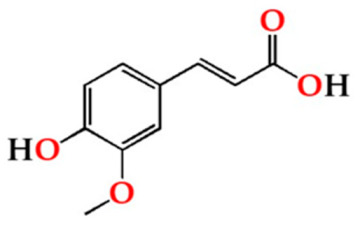	O30	1.70 ± 0.15 Ab	1.71 ± 0.21 Ab	0.46 ± 0.08 Aa	
	O60	1.01 ± 0.35 Ab	1.16 ± 0.23 Ab	0.43 ± 0.04 Aa	
	N	1.08 ± 0.76 Aa	1.31 ± 0.65 Aa	0.33 ± 0.02 Aa	
Syrg	O15	12.66 ± 0.90 Ba	12.78 ± 0.55 Ca	13.12 ± 1.49 Aa	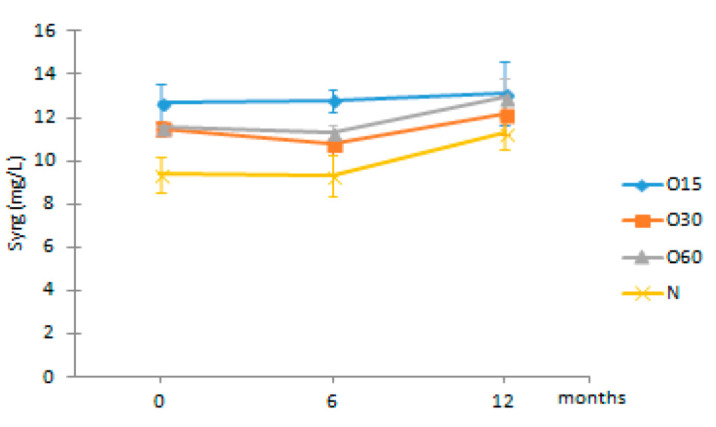
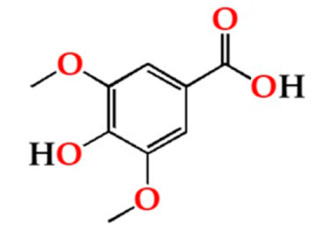	O30	11.50 ± 0.35 ABa	10.77 ± 0.52 Ba	12.17 ± 0.92 Aa	
	O60	11.58 ± 0.13 ABa	11.29 ± 0.37 Ba	12.97 ± 0.85 Aa	
	N	9.38 ± 0.83 Aa	9.31 ± 0.95 Aa	11.27 ± 0.72 Aa	

Results are expressed as mean values ± standard deviation (*n* = 4). For compound: different uppercase letters (A, B, C) in the same column denote significant differences between ageing modalities in each storage time by Tukey’s test (*p* < 0.05); different lowercase letters (a, b) in the same row denote significant differences between storage times for each ageing modality by Tukey’s test (*p* < 0.05). Gall—gallic acid; Van—vanillic acid; Ellag—ellagic acid; Fer—ferulic acid; Syrg—syringic acid.

**Table 2:** Evolution of the phenolic aldehydes from aged WS in each storage time (0, 6, 12 months) according to ageing modalities (O15, O30, O60 and N).

	Time (Months)				
Phenolic Aldehydes (mg/L)	MOX	0	6	12	
Vanil	O15	6.05 ± 0.27 Aa	6.45 ± 0.64 Ba	6.52 ± 0.20 Ba	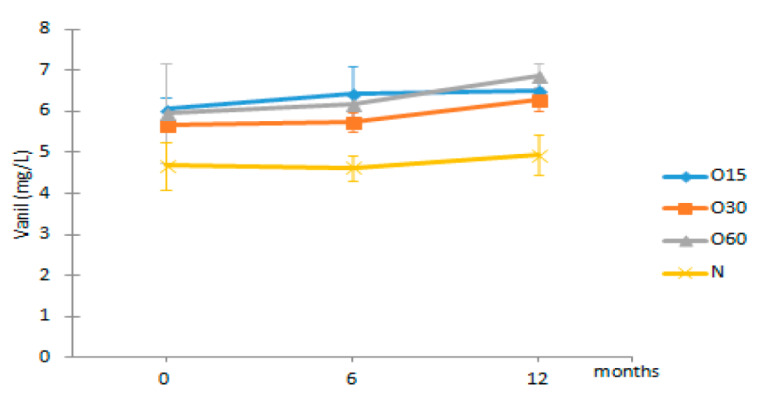
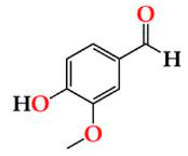	O30	5.66 ± 0.03 Aa	5.74 ± 0.25 Ba	6.29 ± 0.28 Ba	
	O60	5.97 ± 1.21 Aa	6.19 ± 0.17 Ba	6.88 ± 0.29 Ba	
	N	4.68 ± 0.59 Aa	4.62 ± 0.31 Aa	4.93 ± 0.50 Aa	
Syrde	O15	16.01 ± 0.17 Aa	16.80 ± 1.32 Ca	17.20 ± 0.11 Ba	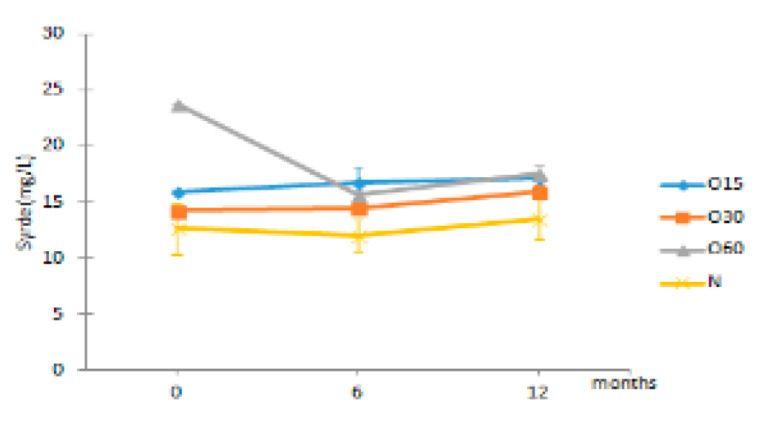
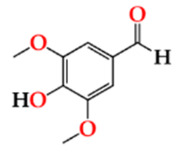	O30	14.32 ± 0.35 Aa	14.49 ± 0.26 Ba	16.01 ± 0.49 Bb	
	O60	23.71 ± 11.25 Aa	15.67 ± 0.26 BCa	17.50 ± 0.38 Ba	
	N	12.65 ± 2.30 Aa	12.05 ± 1.38 Aa	13.52 ± 1.89 Aa	
Cofde	O15	5.74 ± 0.47 Aa	5.30 ± 0.66 Aa	5.81 ± 0.23 Aa	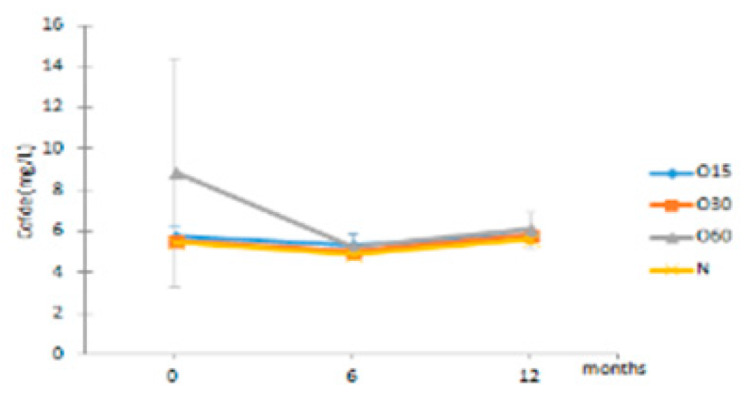
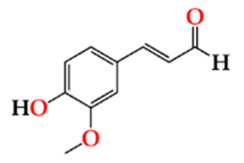	O30	5.54 ± 0.25 Aa	5.01 ± 0.29 Aa	5.81 ± 0.31 Aa	
	O60	8.87 ± 5.58 Aa	5.25 ± 0.63 Aa	6.09 ± 0.89 Aa	
	N	5.43 ± 0.13 Ab	4.88 ± 0.28 Aa	5.62 ± 0.26 Ab	
Sipde	O15	27.48 ± 0.78 Aa	24.59 ± 2.13 Ba	25.45 ± 0.39 Aa	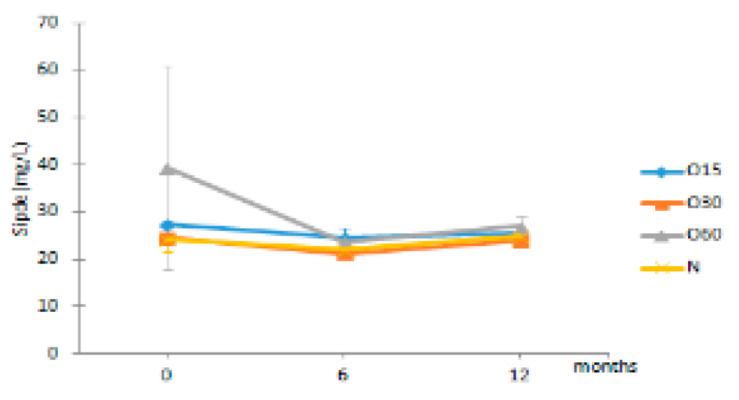
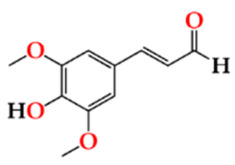	O30	24.61 ± 0.17 Ab	21.31 ± 0.54 Aa	23.98 ± 0.85 Ab	
	O60	39.53 ± 21.54 Aa	23.69 ± 1.30 ABa	27.06 ± 2.25 Aa	
	N	24.43 ± 2.65 Aa	22.08 ± 1.29 ABa	24.96 ± 1.40 Aa	

Results are expressed as mean values ± standard deviation (*n* = 4). For compound: different uppercase letters (A, B, C) in the same column denote significant differences between ageing modalities in each storage time by Tukey’s test (*p* < 0.05); different lowercase letters (a, b) in the same row denote significant differences between storage times for each ageing modality by Tukey’s test (*p* < 0.05). Vanil—vanillin, Syrde—syringaldehyde, Cofde—coniferaldehyde; Sipde—sinapaldehyde.

**Table 3:** Evolution of the furanic aldehydes from aged WS in each storage time (0, 6, 12 months) according to ageing modalities (O15, O30, O60 and N).

	Time (Months)				
Furanic Aldehydes (mg/L)	MOX	0	6	12	
Furf	O15	74.32 ± 7.40 Aa	72.36 ± 4.87 Ba	72.80 ± 5.83 Ba	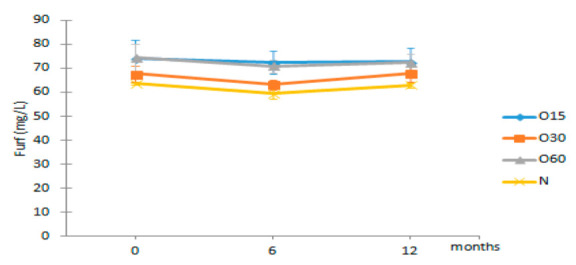
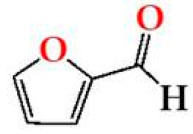	O30	67.70 ± 3.30 Aa	63.21 ± 2.06 Aa	67.94 ± 3.72 ABa	
	O60	74.34 ± 5.64 Aa	70.89 ± 2.68 Ba	72.42 ± 3.56 Ba	
	N	63.63 ± 0.35 Ab	59.42 ± 2.21 Aa	62.95 ± 0.97 Ab	
HMF	O15	33.55 ± 14.11 Aa	31.60 ± 10.16 Aa	33.38 ± 11.36 Aa	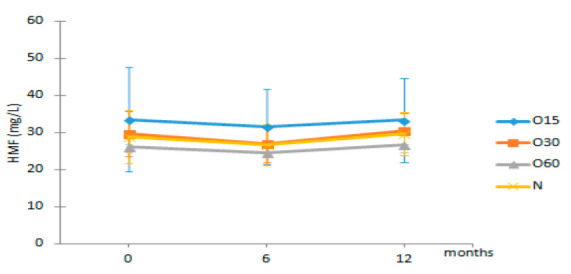
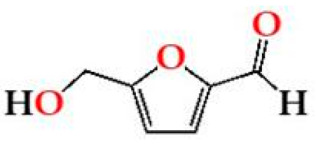	O30	29.64 ± 6.00 Aa	26.95 ± 5.02 Aa	30.41 ± 4.68 Aa	
	O60	26.08 ± 2.69 Aa	24.53 ± 1.12 Aa	26.63 ± 1.63 Aa	
	N	28.91 ± 7.13 Aa	26.71 ± 5.58 Aa	29.72 ± 5.80 Aa	
5Mfurf	O15	1.96 ± 0.31 Ab	0.74 ± 0.28 Aa	0.82 ± 0.23 Aa	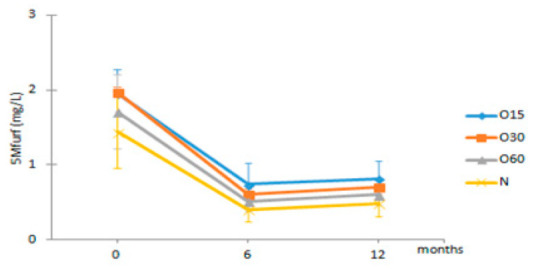
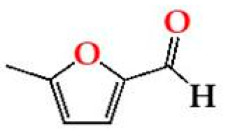	O30	1.97 ± 0.07 Ab	0.60 ± 0.08 Aa	0.71 ± 0.10 Aa	
	O60	1.71 ± 0.49 Ab	0.51 ± 0.13 Aa	0.60 ± 0.15 Aa	
	N	1.44 ± 0.48 Ab	0.40 ± 0.16 Aa	0.48 ± 0.17 Aa	

Results are expressed as mean values ± standard deviation (*n* = 4). For compound: different uppercase letters (A, B) in the same column denote significant differences between ageing modalities in each storage time by Tukey’s test (*p* < 0.05); different lowercase letters (a, b) in the same row denote significant differences between storage times for each ageing modality by Tukey’s test (*p* < 0.05). Furf—furfural; HMF—5-hydroxymethylfurfural; 5Mfurf—5-methylfurfural.

**Table 4:** Description of each ageing technology and the storage in bottle.

Ageing Trial			Storage in Bottle	
Ageing Technology	MOX Flow Rate	N_2_ Flow Rate	Storage Time	Samples Code
O15	2 mL/L/month–0 to15th day 0.6 mL/L/month–15 to 365th day	−	0 months 6 months 12 months	O151_G0a_, O151_G0b_; O152_G0a_, O152_G0b_ O151_G6a_, O151_G6b_; O152_G6a_, O152_G6b_ O151_G12a_, O151_G12b_; O152_G12a_, O152_G12b_
O30	2 mL/L/month–0 to 30th day 0.6 mL/L/month–30 to 365th day	−	0 months 6 months 12 months	O301_G0a_, O301_G0b_; O302_G0a_, O302_G0b_ O301_G6a_, O301_G6b_; O302_G6a_, O302_G6b_ O301_G12a_, O301_G12b_; O302_G12a_, O302_G12b_
O60	2 mL/L/month–0 to 60th day 0.6 mL/L/month–60 to 365th day	−	0 months 6 months 12 months	O601_G0a_, O601_G0b_; O602_G0a_, O602_G0b_ O601_G6a_, O601_G6b_; O602_G6a_, O602_G6b_ O601_G12a_, O601_G12b_; O602_G12a_, O602_G12b_
N	−	20 mL/L/month 0 to 365th day	0 months 6 months 12 months	N1_G0a_, N1_G0b_; N2_G0a_, N2_G0b_ N1_G6a_, N1_G6b_; N2_G6a_, N2_G6b_ N1_G12a_, N1_G12b_; N2_G12a_, N2_G12b_

## Data Availability

The data supporting the findings of this study are available within the article.
